# Fermentation of plant-based milk alternatives for improved flavour and nutritional value

**DOI:** 10.1007/s00253-019-10175-9

**Published:** 2019-11-04

**Authors:** Muzi Tangyu, Jeroen Muller, Christoph J. Bolten, Christoph Wittmann

**Affiliations:** 1 Institute of Systems Biotechnology, Saarland University, Campus A1.5, 66123 Saarbrücken, Germany; 2 Institute of Material Sciences, Department of Biology, Nestlé Research, Lausanne, Switzerland

**Keywords:** Milk alternative, Plant-based, Nutrition, Mixed-culture, Synergistic, Lactic acid bacteria, Soymilk, Plant processing, Systems biology, Vitamin, L-lysine, Anti-nutrients

## Abstract

Non-dairy milk alternatives (or milk analogues) are water extracts of plants and have become increasingly popular for human nutrition. Over the years, the global market for these products has become a multi-billion dollar business and will reach a value of approximately 26 billion USD within the next 5 years. Moreover, many consumers demand plant-based milk alternatives for sustainability, health-related, lifestyle and dietary reasons, resulting in an abundance of products based on nuts, seeds or beans. Unfortunately, plant-based milk alternatives are often nutritionally unbalanced, and their flavour profiles limit their acceptance. With the goal of producing more valuable and tasty products, fermentation can help to the improve sensory profiles, nutritional properties, texture and microbial safety of plant-based milk alternatives so that the amendment with additional ingredients, often perceived as artificial, can be avoided. To date, plant-based milk fermentation mainly uses mono-cultures of microbes, such as lactic acid bacteria, bacilli and yeasts, for this purpose. More recently, new concepts have proposed mixed-culture fermentations with two or more microbial species. These approaches promise synergistic effects to enhance the fermentation process and improve the quality of the final products. Here, we review the plant-based milk market, including nutritional, sensory and manufacturing aspects. In addition, we provide an overview of the state-of-the-art fermentation of plant materials using mono- and mixed-cultures. Due to the rapid progress in this field, we can expect well-balanced and naturally fermented plant-based milk alternatives in the coming years.

## Introduction

**Fig. 1:**
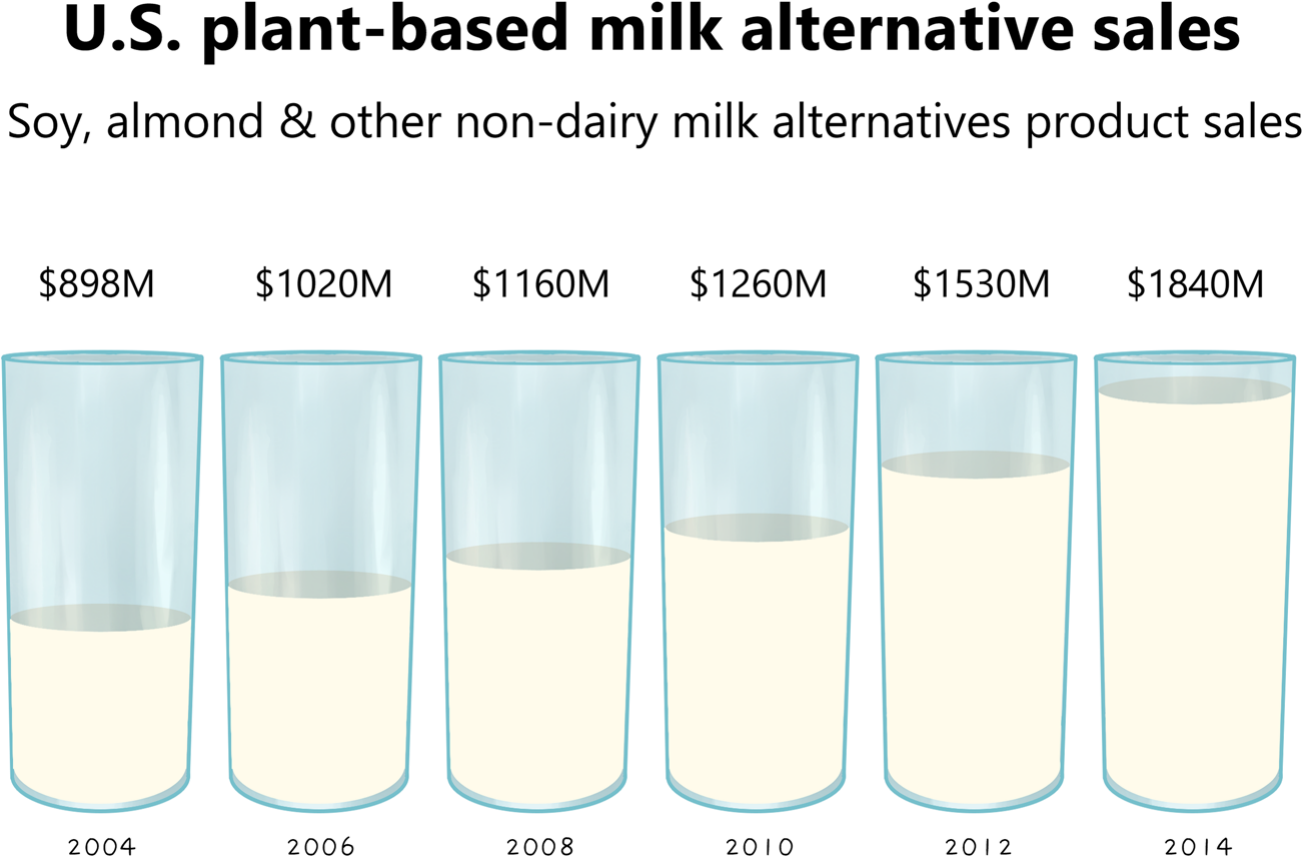
U.S. market development for plant-based milk alternative products (soy, almond and other non-dairy milk products). The data are taken from Bloomberg Surveillance (2015)

Plant-based milk alternatives have been consumed for hundreds and thousands of years. These products are meant to resemble animal milk, an emulsion containing nutrients such as lipids, proteins, amino acids, vitamins and minerals and produced by lactating mammals to provide nutrients for the growth and development of their sucklings (Haug et al. 2007; Mäkinen et al. 2016; Sethi et al. 2016). Today, milk alternatives are commercially obtained from a variety of plants, such as legumes, seeds, nuts, cereals and pseudo-cereals (Mäkinen et al. 2016). Over the past years, the market for these plant-based milk alternatives has continually increased and, in the USA alone, reached an annual volume of approximately US$1.8 billion (Fig. 1). From a global perspective, the projected compound annual growth rate (CAGR) is higher than 10%, and thus the world market is estimated to surpass US$26 billion by 2023 (Bloomberg Surveillance 2015). The increasing preference for plant-based milk alternatives is driven by different factors and consumer demands: health-related challenges such as lactose intolerance and milk allergies (Crittenden and Bennett 2005), consumer concerns about cow milk hormones and cholesterol (Epstein 1990), ethical disputes regarding the use of animals (Hughes 1995), environmental issues (Rotz et al. 2010), changes in lifestyle towards vegetarian and vegan food, presumably healthier diet (Craig 2010) and the marketed health-promoting properties of these products (Paucar-Menacho et al. 2010). Accordingly, leading dairy companies are adding plant-based milk alternative products to their portfolio.

Plant-based milk alternatives are intended to resemble animal milk in terms of colour and texture (Mäkinen et al. 2016; Sethi et al. 2016). However, they often do not provide the full nutritional value of cow milk (Sethi et al. 2016) and suffer from undesirable off-flavours (Desai et al. 2002; Sethi et al. 2016; Vanga and Raghavan 2018). Therefore, commercial products positioned as plant-based milk alternatives are typically amended with additives such as vitamins, amino acids, and minerals (Sethi et al. 2016). However, leading food and beverage companies have committed to removing ingredients perceived as artificial from their products—clean label foods and beverages are not only a trend but are seemingly becoming an expectation. Accordingly, natural plant-based milk alternatives, which meet the nutritional quality and taste of animal-derived milk without blending, are of particular interest (Asioli et al. 2017).

An appealing option to reach this goal is fermentation. Since the early days of mankind, fermentation has been a natural approach to produce food, and today, fermented foods are more popular than ever before (Adler et al. 2013). During the production of coffee, bread, chocolate, wine, cheese, mixed pickles, kombucha, kimchi, and sauerkraut, food-grade microbes improve the nutritional value, aroma and taste, texture and stability of foods and beverages and contribute to their microbial safety. In particular, the application of mono-culture fermentation to food products is well understood (Leroy and De Vuyst 2004; National Research Council 1992). More recently, the design of mixed-culture fermentation with two or more microorganisms, naturally occurring in many food production processes (Adler et al. 2013), is becoming increasingly important (Ciani et al. 2010; Smid and Lacroix 2013). The latter appears particularly promising for plant-based milk alternative fermentation due to the potential synergistic effects within the microbial consortia, which helps to improve quite diverse quality criteria with only one process (National Research Council 1992; Sieuwerts et al. 2008).

In this regard, this review introduces the key criteria for major plant-based milk alternatives, including nutritional and sensory qualities as well as manufacturing perspectives. In relevant examples, mono- and mixed-culture fermentations of plant-based milk alternatives are presented to highlight state-of-the-art and future avenues for research and development.

## Leading plant-based milk alternatives

### Plant types

Due to a constantly increasing demand for non-dairy alternatives and growing interest in exploring different functional properties, various plants have been used to produce non-dairy milk alternatives (Sethi et al. 2016). The relevant plant sources can be classified into five types: (i) legumes (beans), (ii) nuts, (iii) seeds, (iv) pseudo-cereals, and (v) cereals (Fig. 3) (Sethi et al. 2016). Soy-based drinks are the dominant plant-based milk alternatives in the Western world (Mäkinen et al. 2016). In addition, drinks based on almond (Ginsberg and Ostrowski 2007), coconut (Seow and Gwee 1997), sunflower seed (Fujisawa et al. 1986), chickpea (Rao et al. 1988), lupine (Ivanović et al. 1983), hemp (Vahanvaty 2009), sesame (Afaneh et al. 2011), quinoa (Pineli et al. 2015), pea (Li et al. 2004), and rice (Mitchell et al. 1990) are available and contribute to the diversity of the plant-based milk alternative market. Depending on the individual raw materials, the corresponding drinks differ significantly in composition and flavour.

### Key quality criteria

**Fig. 2:**
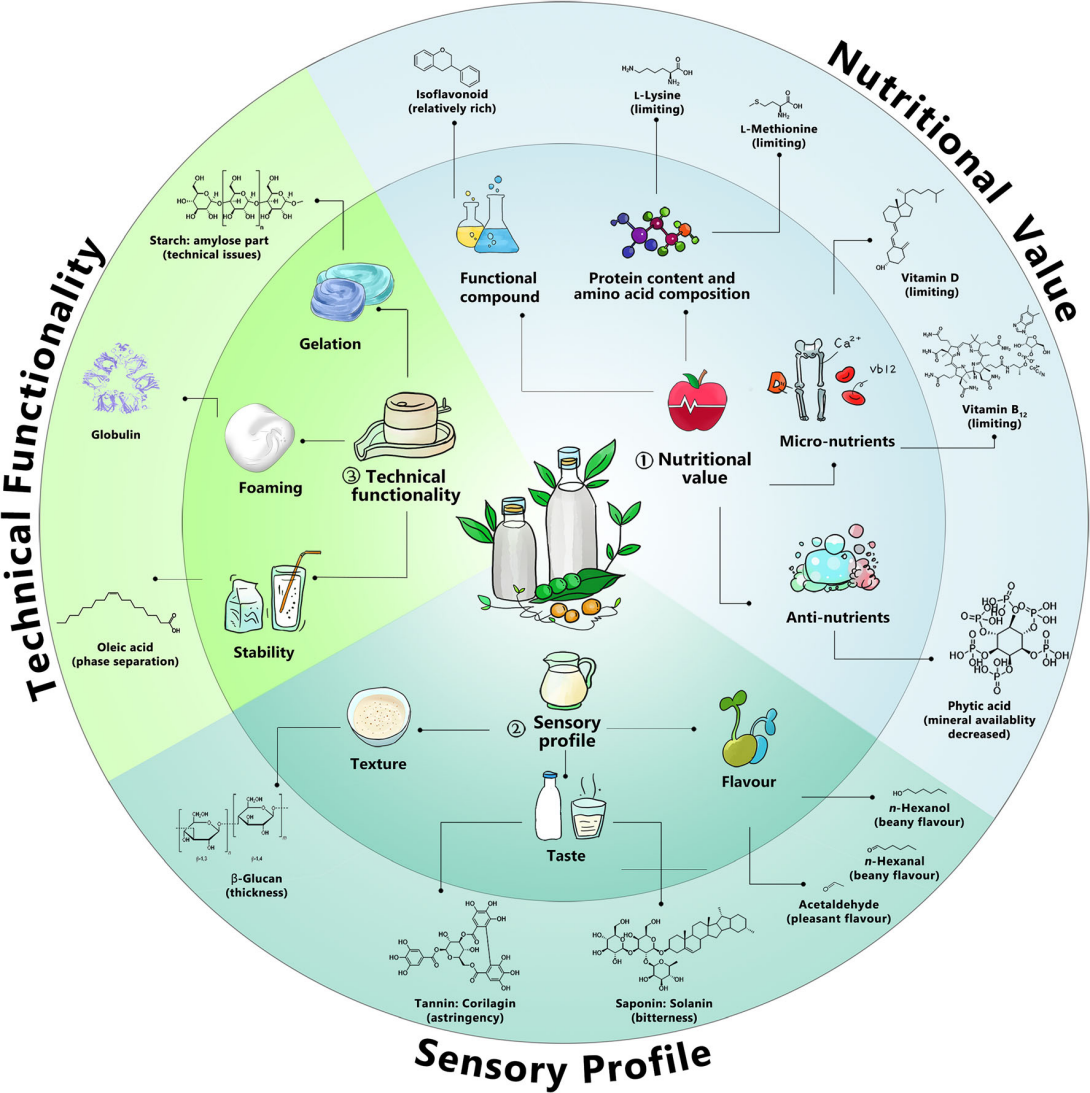
Quality criteria of plant-based milk alternatives

**Fig. 3:**
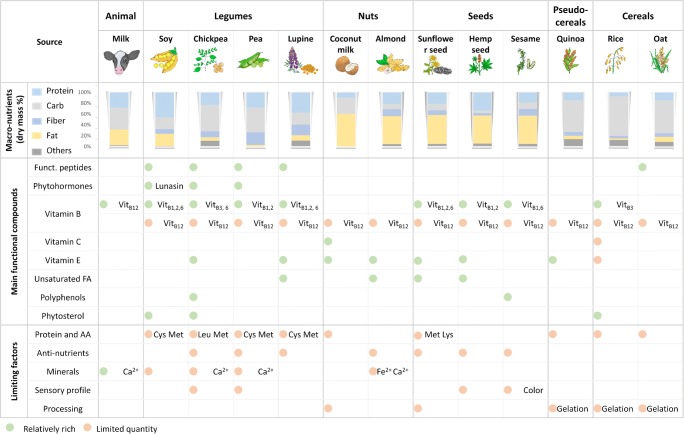
Macronutrient composition, functional components and limiting factors of common plants used for plant-based milk alternative production. The data are collected from previous work (Afaneh et al. 2011; Bernat et al. 2015; Callaway 2004; DebMandal and Mandal 2011; Duranti et al. 2008; Erbaş et al. 2005; Fernandez and Berry 1988; Hove 1974; Juliano and Hicks 1996; Lambo et al. 2005; Lampart-Szczapa et al. 2003; Lebiedzińska and Szefer 2006; Makinde and Akinoso 2013; Moneret-Vautrin et al. 1999; Noimark and Cox 2008; Önning et al. 1998; Paucar-Menacho et al. 2010; Ranhotra et al. 1993; Roy et al. 2010; Seow and Gwee 1997; Sethi et al. 2016; Škrbić and Filipčev 2008; Ulyatu et al. 2015; Vahanvaty 2009; Vanga and Raghavan 2018; Vidal-Valverde et al. 2003; Vilche et al. 2003; Villamide and San Juan 1998; Wood and Grusak 2007). The micronutrient composition data are acquired from the National Nutrient Database for Standard Reference Release (NDB) (https://ndb.nal.usda.gov/ndb/). The NDB identification of the selected materials is as follows: milk (01212), soy (16111), chickpea (45041830), pea (45272128), lupine (16076), coconut milk (45117929), almond (12061), sunflower seed kernels (12036), hemp seed (12012), sesame seed (12023), quinoa (20035), rice (20090) and oat (20132). carb, carbohydrates except fibre; funct. peptides, functional peptides; unsaturated FA, unsaturated fatty acids.

Plant-based milk alternatives should preferably resemble the technical, nutritional and organoleptic properties of cow milk. To achieve this goal, researchers and developers in academia and industry must overcome certain challenges (Figs. 2 and 3).

### Physico-chemical properties

**Fig. 4:**
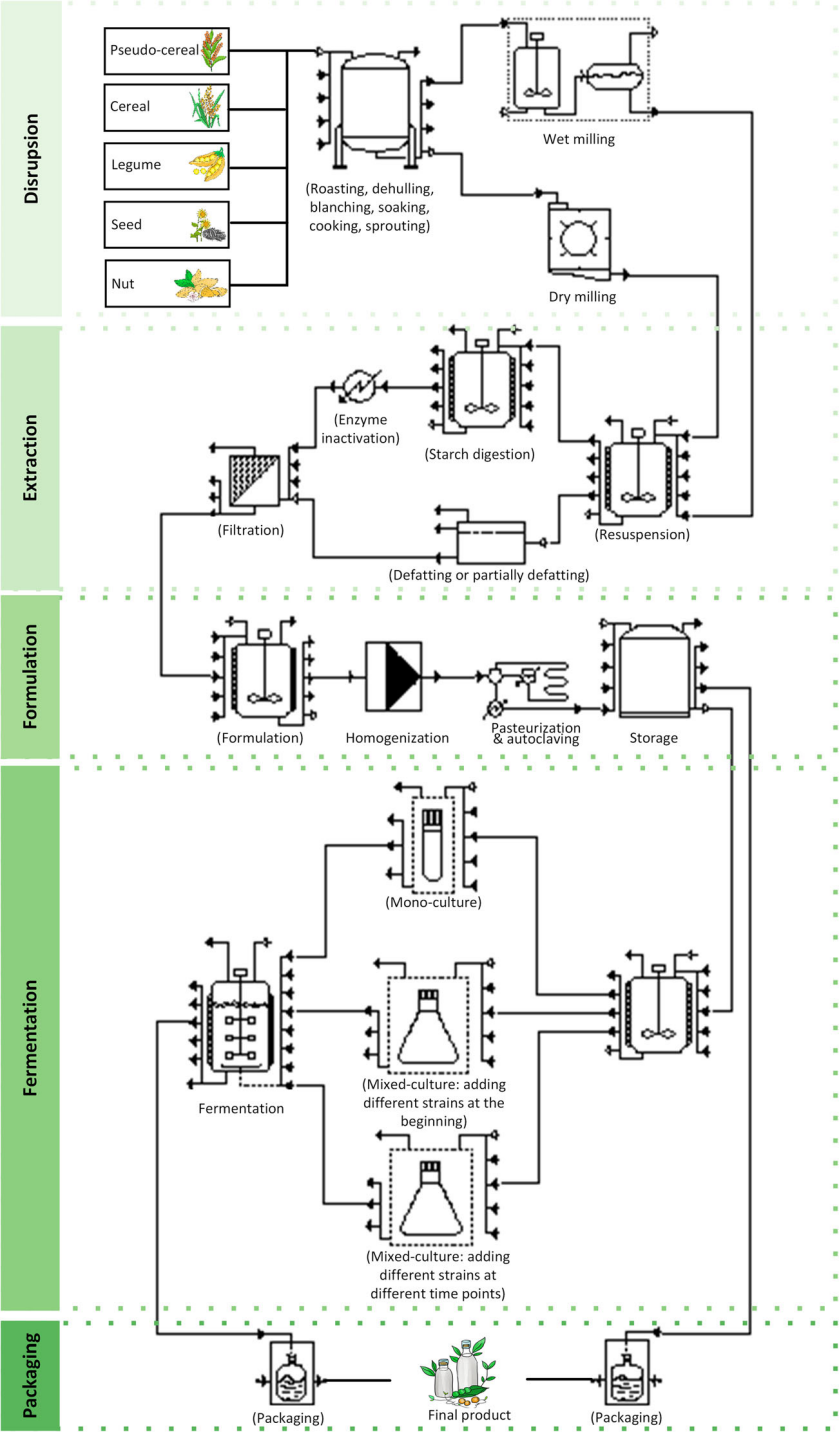
Flow chart for the manufacturing of plant-based milk alternatives. The unit operations given in brackets are optional and depend on the chosen raw material and the desired quality of the final product

Plant-based milk alternative manufacturing generally employs consecutive unit operations (Fig. 4). Generally, plant-based drinks are prepared by crushing the plant material, followed by extraction of its soluble parts into water. The properties of the final product depend on the raw material and, furthermore, on the specifications of the individual disintegration, homogenisation, formulation, emulsification, and storage processes. Different strategies are applied to make the homogenisation and stability of plant-based milks more similar to that of animal milk, which is a natural emulsion. For example, plant-based drinks from starchy materials (such as cereals or pseudo-cereals) easily gelate during sterilisation (autoclaving or pasteurisation), which causes technical problems in downstream processing (Mäkinen et al. 2016). Furthermore, the excessive lipid content of seeds and nuts may lead to an undesired phase separation and reduced product stability (Figs. 2 and 3) so that these compounds are removed during processing (Briviba et al. 2016). More details on plant-based milk alternative manufacturing can be found in an excellent recent review (Mäkinen et al. 2016).

### Nutritional value and bioactive components

Without doubt, the plants used offer certain attractive properties (Fig. 3). Some of the raw materials, such as legumes and seeds, have a protein content comparable to that of cow milk (although the amino acid quality is not comparable to the same extent). Moreover, the raw materials are rich in certain micronutrients (vitamin, minerals) (Gernand et al. 2016) and contain bioactive compounds such as antioxidants (Zhao and Shah 2014), dietary fibres (Kohajdová et al. 2006) and phytoestrogens (Verdeal and Ryan 1979) (Figs. 2 and 3). The latter contributes to health benefits, including a lowered risk of osteoporosis, heart disease, breast cancer, and menopausal symptoms (Patisaul and Jefferson 2010). However, phytohormones potentially also cause adverse health effects, which may depend on human age, health status and even the presence or absence of specific gut microflora, so that this area requires more research in the future (Patisaul and Jefferson 2010).

In recent years, several interesting studies have investigated the presence and impact of micro-nutrients and bioactive compounds in plant materials. As an example, almond, peanut, and coconut exhibit significant amounts of vitamins E and C, which confer antioxidant properties (Sethi et al. 2016). Legumes are a good source for essential mono- and polyunsaturated fatty acids, minerals (Fe^2+^, Zn^2+^, Mg^2+^) (Sandberg 2002), and phytoestrogens (isoflavones) (Pyo et al. 2005) (Figs. 2 and 3). In other types of plant materials, β-glucans contribute to health benefits (in lowering cholesterol levels) and increase the sensory attributes of the final products (Lazaridou and Biliaderis 2007; Othman et al. 2011).

Despite these undoubted beneficial properties of plant raw materials, a careful inspection, however, reveals that commercial plant-based milk alternatives are not nutritionally balanced and comparable to animal milk. In particular, the protein content of plant-based drinks can be low. Approximately 50% of commercial plant-based milk alternatives contain little or even no protein (< 0.5%), while only selected soy-based milk analogues reach the higher protein level of cow milk (3.7%) (Jeske et al. 2017). Additionally, plant proteins often exhibit low quality, poor digestibility and an undesired limitation in essential amino acids (Millward 1999). l-Lysine, l-methionine, l-cysteine and l-tryptophan are amino acids that are typically underrepresented (Millward 1999). In addition, certain vitamins, such as vitamin D and vitamin B_12_, are present at low levels or are even absent (Table 1), which may be part of the reason for the vitamin deficiency of people following a strict vegetarian diet (Pawlak et al. 2014). Moreover, vitamins are sensitive molecules and some of them are easily degraded during washing and heating, which further reduces their content (Fig. 4). Other important compounds suffer from low bioavailability. For instance, soy isoflavones mainly exist in the form of genistin and daidzin, which are glucosides of genistein and daidzein and far less bioavailable than the corresponding aglycone forms (Vacek et al. 2008; Xu et al. 1994). Moreover, plant-derived products can contain anti-nutritional factors. For example, phytates and saponins form insoluble complexes with valuable minerals (such as Ca^2+^, Mg^2+^, Fe^2+^ and Zn^2+^), which decrease their bioavailability (Rekha and Vijayalakshmi 2010; West et al. 1978). Plant-based oligosaccharides, such as raffinose, stachyose, and verbascose, can only be digested by intestinal bacteria through fermentation, which results in flatulence, diarrhoea, and other discomforts (Onyesom et al. 2005). Furthermore, the intestinal tract can be disturbed by trypsin and other protease inhibitors in plant-based milk alternatives, which interfere with protein and starch digestion by inactivating the digesting enzymes (Anderson and Wolf 1995).

**Table 1:** Nutritional comparison of cow milk and selected plant materials used for the production of plant-based milk alternatives

	Cow milk (dry)	Soybean (dry)	Sunflower seed (dry, partially defatted)	Oat (dry, partially debranned)
Protein (g/100 g)	26	43	48	15
Ca^2+^ (mg/100 g)	912	140	114	55
Fe^2+^ (mg/100 g)	0.47	3.95	6.62	4.00
Vitamin B_1_ (thiamine) (mg/100 g)	0.28	0.43	3.19	0.69
Vitamin B_2_ (riboflavin) (mg/100 g)	1.20	0.76	0.27	0.12
Vitamin B_3_ (niacin) (mg/100 g)	0.65	1.06	7.31	1.47
Vitamin B_6_ (mg/100 g)	0.30	0.22	0.75	0.12
Total folate (μg/100 g)	37	205	222	32
Vitamin B_12_ (μg/100 g)	3.2	0.0	0.0	0.0
Vitamin D (D_2_ + D_3_) (μg/100 g)	0.5	0.0	0.0	0.0

The data are taken from the USDA National Nutrient Database for Standard Reference (https://ndb.nal.usda.gov/ndb/)

### Sensory profile

It has been shown by consumer and marketing studies that taste has a key impact on food selection (Glanz et al. 1998). In this regard, the natural taste of plant-based milk alternatives, unfortunately, exhibits only limited acceptance (Mäkinen et al. 2016). Although certain components of plant materials (such as soluble fibres) positively influence texture and mouthfeel (Vasquez-Orejarena et al. 2018), plant-based milk alternatives are still generally perceived as products with a displeasing taste, probably also because of previous experiences with less appealing products in the market (Wansink et al. 2005). Legume-based products tend to smell beany and earthy, which is considered undesirable in countries without traditional consumption of these types of products. Volatile compounds such as *n*-hexanal and *n*-hexanol, which originate from the oxidation of plant lipids, are mainly responsible for this type of off-flavour. Plant phenols (including anti-nutrients such as tannins and saponins), terpenes, glucosinolates, and flavonoids impart bitter, acrid or astringent tastes, depending on their molecular weights (Drewnowski and Gomez-Carneros 2000). Regrettably, certain bioactive (and therefore otherwise beneficial) compounds such as isoflavonoids are also linked to an objectionable aftertaste (Matsuura et al. 1989). Additionally, a greenish, greyish or brownish colour, which corresponds to the colour of the raw plant material; a chalky or sandy texture; and a thin mouthfeel due to the presence of insoluble particles negatively influence consumer purchase willingness (Peyer et al. 2016).

### Technical processing and fortification

To solve some of the abovementioned challenges, different manufacturing strategies have been developed. In early processing steps, excess lipids (from nuts and seeds) and excess starch (from cereals and pseudo-cereals) are separated and/or enzymatically hydrolysed to prevent phase separation and gelation and increase product stability (Rustom et al. 1993). Homogenisation is used to disrupt larger particles and lipid droplets and achieve uniform particle size, which also improves product stability (Briviba et al. 2016).

To overcome the known nutritional and sensory limitations, commercial plant-based milk alternatives are typically supplemented with sweeteners, artificial flavours, protein, amino acids, minerals (Ca^2+^, Mg^2+^, Fe^2+^ and Zn^2+^), and vitamins (B_12_, B_2_, D and E) (Sethi et al. 2016; Zhang et al. 2007). Moreover, extended mechanical and thermal pre-processing (e.g. roasting, dehulling, blanching, soaking, cooking and sprouting) is applied to reduce anti-nutrients such as protease inhibitors (Jiang et al. 2013; Yuan et al. 2008), decrease and mask off-flavour and improve mouthfeel and colour (Dakwa et al. 2005; Kim et al. 1986). However, some anti-nutrients are very resistant. For example, phytates cannot be destroyed entirely even by heating to 100 °C (Anderson and Wolf 1995).

## Fermentation of plant materials

Fermentation has been applied to cereals such as maize, wheat, rice and sorghum for a long time (National Research Council 1992). Plant materials support the growth of microorganisms (Espirito-Santo et al. 2014; Peyer et al. 2016; Sethi et al. 2016). Lactic acid bacteria (LAB), bacilli and yeasts (e.g. *Saccharomyces*) are the most widely used microbes for plant-based fermentation (Jeske et al. 2018; Steinkraus 1997). Being studied mainly as mono-cultures, these microbes have been proven to possess properties that enhance important nutritional and/or sensory attributes.

### Nutritional value

Most importantly, fermentation can increase protein content by the growth of the fermenting food-grade microbes and by improving plant protein solubility and amino acid composition and availability. As an example, *Bifidobacterium* significantly increased the crude protein content of soy-based drinks (Hou et al. 2000). Moreover, fermentation of soybean meal with *Lactobacillus plantarum* resulted in a beneficial increase in essential amino acids such as l-lysine (Song et al. 2008). Notably, specific microbial strains synthesise vitamins during fermentation (LeBlanc et al. 2013), including vitamin K (Bentley and Meganathan 1982) and vitamins of the B group (LeBlanc et al. 2011). Yeast is well known for its ability to produce vitamin B_2_ (Lindegren 1945). Compared to synthetic fortification, fortification by natural vitamin-producing microorganisms is widely recognised as safer, more natural and more environmentally friendly (LeBlanc et al. 2011).

### Anti-nutrients and mineral availability

Fermentation by itself or combined with other treatments such as cooking, sprouting and soaking can dramatically reduce the level of anti-nutrients such as tannins, phytates and cyanides in plant-based food (Anderson and Wolf 1995; Onyesom et al. 2005; Soetan and Oyewole 2009; Wang et al. 2003). As an example, LAB are capable of producing phytases and provide the optimum pH conditions for these enzymes, which then catalyse the hydrolysis of phytates into *myo*-inositol and phosphate, improve digestibility and increase mineral bioavailability (Rekha and Vijayalakshmi 2010). As an example, fermentation of finger millet significantly reduced various undesired anti-nutrients (phytates, tannins, and trypsin inhibitor) while simultaneously enhancing mineral extractability and digestibility (Antony and Chandra 1998).

### Bioactive components

Fermentation is capable of increasing the concentration or bioaccessibility of functional (bioactive) compounds. The fermentation of soy using bacteria with β-glucosidase ability enables the conversion of glucoside isoflavones into aglycone isoflavones of higher bioactivity and bioaccessibility (Pyo et al. 2005), which has also been observed for seeds of kerandang, a flowering plant belonging to the legume family (Titiek et al. 2013). Correspondingly, *L. plantarum* is able to transform sesaminol triglucoside of sesame milk into bioactive sesaminol aglycone with enhanced radical scavenging activity (Ulyatu et al. 2015). It was also reported that LAB fermentation of soy releases bioactive peptides, which inhibit angiotensin-converting enzymes that are related to the desired antihypertensive effect (Hou et al. 2000).

### Sensory profile

Fermentation can improve the sensory profile of plant-based milk alternatives (Mital and Steinkraus 1979). As an example, microbial fermentation decreased the beany flavour of plant materials, probably due to deprivation of *n*-hexanal and *n*-hexanol (Wang et al. 2003). In addition, fermentation can result in desirable volatile flavours. For example, diacetyl (2,3-butanedione), which provides a nice, butterscotch-like aroma, is emitted during cereal-based fermentation (Peyer et al. 2016). Acetaldehyde, delivering a pungent, fruity (green apple) flavour with sweet notes, increases in concentration in peanut, cereal and soy during fermentation (Horáčková et al. 2015; Lee and Beuchat 1991; Sethi et al. 2016). The flavour and taste of plant-based milk alternatives is also affected by changes in the levels of amino acids (Yamanaka et al. 1970).

## Mixed-culture fermentation can provide synergistic effects to enhance quality

An interesting and obviously important concept is the use of mixed cultures to ferment plant materials. Generally, interactions between microbes in mixed cultures are numerous and complex, for example competition (−/− interaction), mutualism (+/+ interaction), commensalism (+/0 interaction), amensalism (−/0 interaction), and parasitism (+/− interaction) (Sieuwerts et al. 2008). Desired interactions during mixed-culture fermentation are mainly of a mutualistic and commensalistic nature, by which beneficial activities of at least one microbe are promoted (National Research Council 1992).

**Fig. 5:**
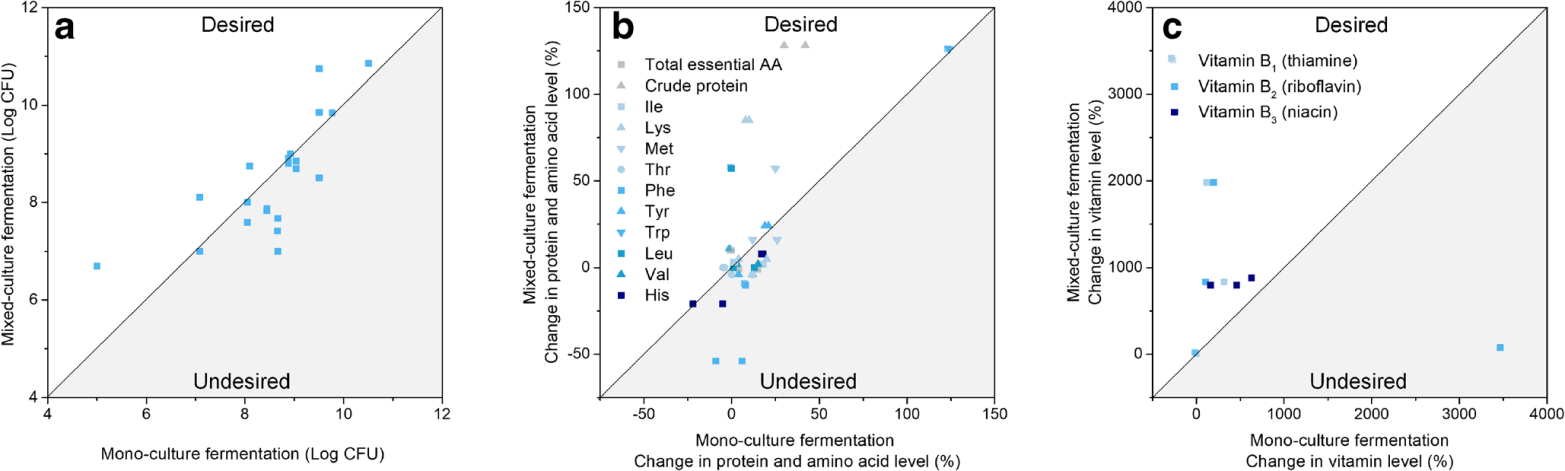
Impact of mixed-culture fermentation on the quality of plant-based milk alternatives. Comparison between mixed-culture and mono-culture fermentation: microbial growth (A), essential amino acid level (B), vitamin level (C). The white area covers desired synergistic effects (desired), while the grey area covers undesired effects that result from mixed-culture fermentation (undesired)

**Fig. 6:**
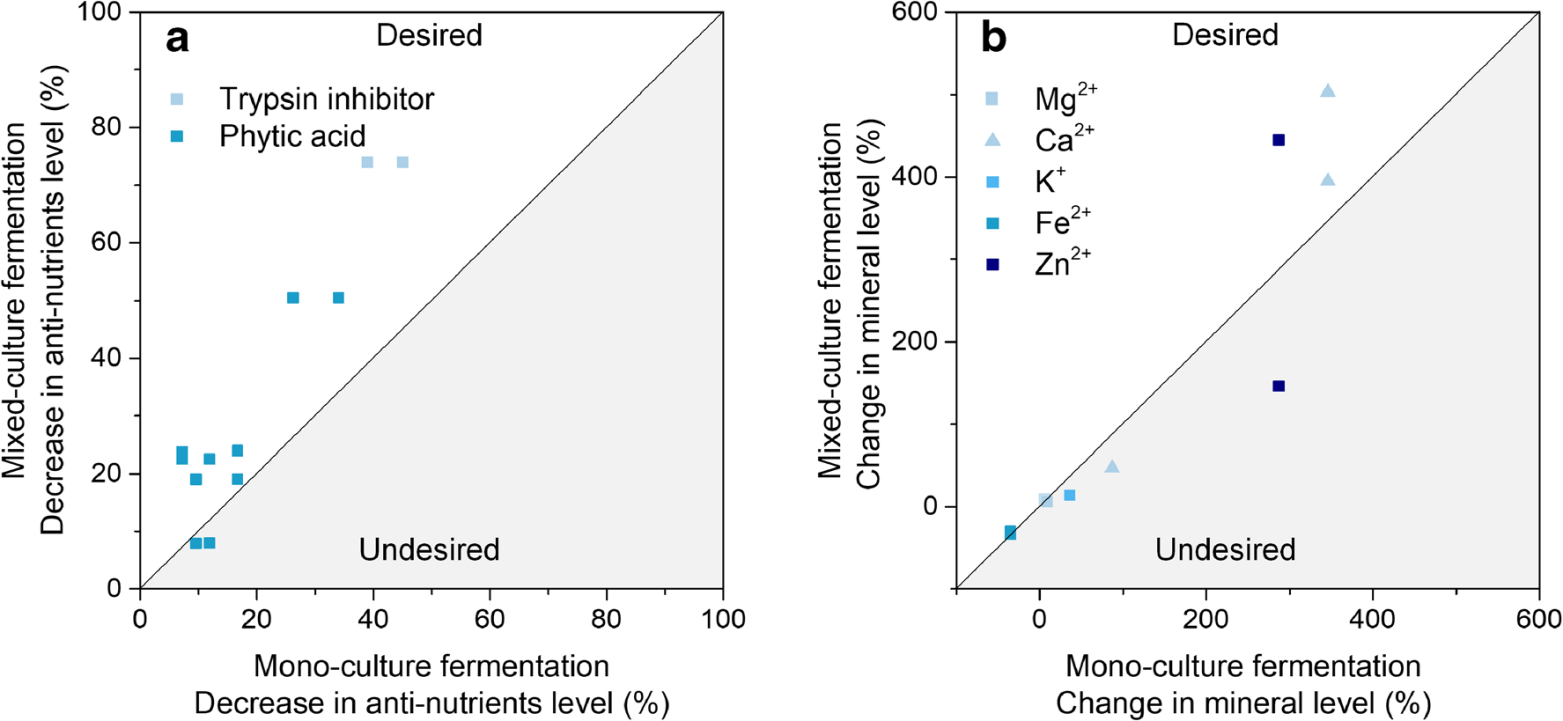
Impact of mixed-culture fermentation on the quality of plant-based milk alternatives. Comparison of the effects of mixed-culture and mono-culture fermentation on the elimination of anti-nutrients (A) and the alteration of mineral contents (B)

The cooperation between *Streptococcus thermophilus* and *Lactobacillus delbrueckii* subsp. *bulgaricus* during yogurt fermentation is a well-understood example of mutualism. The proteolytic *Lactobacillus* strain benefits the non-proteolytic *S. thermophilus* through the release of peptides and free amino acids as a nitrogen source. Conversely, *S. thermophilus* provides *L. delbrueckii* with growth-stimulating factors such as formic acid, pyruvic acid, folic acid and carbon dioxide (Sieuwerts et al. 2008). In a mixed culture, the two strains stimulate one another’s growth, acid production and volatile compound formation (Sieuwerts et al. 2008). A synergistic effect on growth has also been observed for soy fermentation (Chumchuere and Robinson 1999), probably related to the different glycolytic activities of the strains involved. More specifically, microbial consortia can cooperate to enable multistep biotransformations. A prominent example demonstrated a close cooperation of microbes in simulated cocoa pulp fermentation: LAB form lactate, while yeasts form ethanol during the early stage of the fermentation. Both nutrients, in turn, are crucial co-substrates for acetic acid bacteria, which then accumulate acetate, the key molecule that initiates the formation of aroma and flavour compounds (Adler et al. 2013). Demonstrated effects of mixed cultures in the generation of plant-based milk alternatives are summarised in Figs. 5 and 6 and are described in more detail in the following subsections.

### Growth

During soy fermentation, *Lactobacillus fermentum* NRRC 207 and *L. delbrueckii* subsp. *bulgaricus* NCDO 1489 exhibited more than 100-fold higher cell numbers when mixed with *S. thermophilus* than when grown in mono-culture, and both strains improved the growth of *S. thermophilus* as well (Chumchuere and Robinson 1999). Such a synergetic effect was also observed in other studies (Champagne et al. 2009; Mital et al. 1974). The combination of amylolytic and probiotic bacterial strains also reduced the fermentation time of rice because of the resulting elevated acidification rate (Espirito-Santo et al. 2014). In addition, certain yeasts benefit the growth of LAB by excreting specific nutrients (Liu and Tsao 2009; Rekha and Vijayalakshmi 2010). However, not all combinations are desirable for the survival of starter cultures (Angelov et al. 2005). As an example, the viable count of *Bifidobacterium longum* R015 and *L. fermentum* even decreased in specific mixed-culture fermentation (Fig. 5a) (Champagne et al. 2009; Chumchuere and Robinson 1999).

### Nutrient value

The protein content and essential amino acid composition can differ substantially following mono- vs. mixed-culture fermentation of plant-based milk alternatives (Fig. 5b). Co-fermentation of peanut using *Lactobacillus acidophilus* and *L. plantarum* significantly increased the total protein and l-lysine, l-methionine and l-tryptophan contents compared to those of the corresponding mono-culture fermentations (Sanni et al. 1999). Spontaneous co-fermentation of strains originating from cowpea and chickpea improved l-methionine levels by approximately sixfold (Zamora and Fields 1979). However, in other cases, mixed-culture fermentation appeared inferior to mono-culture processes (Santos et al. 2014).

Moreover, mixed cultures can impact vitamin formation. Co-fermentation of *L. plantarum* SM39 and *Propionibacterium freudenreichii* DF13 showed higher levels of folate and vitamin B_12_ and yielded up to 8400 ng/L of folate, which is, otherwise, only achievable with genetically modified strains (Hugenschmidt et al. 2011; Smid and Lacroix 2013). To date, only a few pioneering studies have investigated bacterial vitamin production during plant material fermentation (Fig. 5c). A mixture of *Lactobacillus* strains, including *L. plantarum*, improved the riboflavin, thiamine and niacin contents in cowpea milk compared to the use of mono-cultures (Sanni et al. 1999). Similar effects were observed for soy using a co-fermentation of *Saccharomyces boulardii* and *Lactobacillus casei* (Rekha and Vijayalakshmi 2010). However, many of the tested strain combinations only showed few or even adverse effects in this study (Champagne et al. 2010; Zamora and Fields 1979). It has been speculated that a decrease in a specific vitamin may relate to the fact that the organism itself requires it for growth (Rekha and Vijayalakshmi 2010).

### Anti-nutrients and mineral availability

Interestingly, mixed cultures help to reduce anti-nutrients (Fig. 6a), which, in turn, enhances mineral availability (Fig. 6b). A mixed culture of *L. acidophilus* and *L. plantarum* was more effective than fermentation of the individual strains in eliminating phytic acid and trypsin inhibitors in cowpea (Sanni et al. 1999). Similarly, a mixed culture of *S. thermophilus* CCRC 14085 and *Bifidobacterium infantis* CCRC 14603 dramatically decreased phytic acid (80%) and saponin (30%) levels in soy (Lai et al. 2013). It was further found that a mixed *S. boulardii* and *L. plantarum* B4495 fermentation increased calcium bioavailability approximately sixfold compared to the mono-culture fermentation (Rekha and Vijayalakshmi 2010).

Stachyose and raffinose are undesirable components of plant-based milk alternatives, especially legume-based products, that are linked to flatulence (Desai et al. 2002). Generally, a combination of different strains was more efficient in degrading these carbohydrates than were pure cultures (Santos et al. 2014). Soy fermented by a mixed culture of different LAB yielded a lower level of stachyose and raffinose and a desirable higher content of acetic acid, fructose, glucose and galactose (Wang et al. 2003). Similar effects were also observed in other studies (Santos et al. 2014). However, these outcomes are strongly dependent on the exact strain combination. As an example, *Streptococcus* induced adverse effects in *L. fermentum* and *Bifidobacterium longum* to metabolise stachyose (Champagne et al. 2009; Chumchuere and Robinson 1999).

### Bioactive components

Fermentation of soy by bacteria that possess β-glucosidase activity enables biotransformation of isoflavones into the more bioactive aglycone form (Pyo et al. 2005). Mixed cultures of *S. boulardii* together with five *Lactobacillus* species converted over 95% of the glucoside into the aglycone isoflavone (Rekha and Vijayalakshmi 2010). However, other strain mixtures revealed lower bioconversion efficiency compared to that of pure cultures. For example, a weaker bioconversion was detected when *S. thermophilus* was mixed with *B. infantis*, *B. longum* and *Lactobacillus helveticus* (Champagne et al. 2010; Chien et al. 2006). This observation emphasises again the importance of strain selection in mediating the synergetic effects between the different cultures.

### Sensory values

Although mono-culture fermentation seems to be as efficient as mixed-culture fermentation in lowering the content of the off-flavour molecules *n*-hexanal and *n*-hexanol (Lee 2001), mixed-culture fermentation appears to be more useful in generating preferred flavour enhancers. For example, acetaldehyde, a key compound of the desired yogurt flavour, is formed more extensively by a mixture of two or more cultures (Horáčková et al. 2015; Lee 2001; Liu et al. 2002). A mixed culture of *L. delbrueckii* subsp. *bulgaricus* and *Streptococcus salivarius* subsp. *thermophilus* not only decreased the beany flavour of peanut milk but also significantly increased whiteness, viscosity, gumminess and smoothness (Lee 2001). An increase in luminosity and whiteness index values was also observed following almond fermentation by a mixed culture of *Lactobacillus reuteri* and *S. thermophilus* (Bernat et al. 2015).

## Conclusion

The market for plant-based milk alternatives is quickly increasing. However, the unbalanced nutrition and unwanted organoleptic characteristics of these products still limit their consumption. The use of mixed-culture fermentation, in particular, holds great potential for improving the nutritional quality and the sensory profile of plant materials. Previous studies clearly show that the performance of mixed cultures is strongly species- and strain-dependent. At present, strain combination is still conducted with trial and error approaches. It seems difficult, if not even infeasible, to predict the effects of a mixed culture due to our still poor understanding of the underlying microbial interactions involved. Possibilities for a more rational selection and combination of strains with predictable synergistic interactions would be highly valuable towards developing smarter fermentation processes and better products.

Recently, systems biology approaches have greatly advanced and opened up novel possibilities to study even complex systems with a great level of detail. Due to the enormous progress in the field, quantitative systems biology studies of mixed-culture fermentations of plant-based milk alternatives could become a next level of research to better understand the underlying physiological, cellular and molecular processes. The system to be studied is admittedly complex, but seminal studies on similarly complex fermentation processes involving cocoa fermentation (Adler et al. 2013), oil-based riboflavin production (Schwechheimer et al. 2018), growth on substrate mixtures (Schilling et al. 2007) and under environmental changes (Hou et al. 2000; Kohajdová et al. 2006; Kohlstedt et al. 2014; Wittmann et al. 2007) are encouraging success stories, which demonstrate the power of systems biology to shed more light on this subject and provide valuable guidance for improvement. It can be expected that similar systems level studies, which unravel genomics, transcriptomics, proteomics, metabolomics, and fluxomics in multi-omics approaches, will significantly contribute to a better understanding of plant material fermentation and advance rational designs and improvements in this field. In addition, as the market becomes increasingly diverse, the fermentation of novel types of plant materials will become another important trend.
